# The Complex Structure of the Pharmacological Drug–Disease Network

**DOI:** 10.3390/e23091139

**Published:** 2021-08-31

**Authors:** Irene López-Rodríguez, Cesár F. Reyes-Manzano, Ariel Guzmán-Vargas, Lev Guzmán-Vargas

**Affiliations:** 1 Laboratorio de Sistemas Complejos, Unidad Interdisciplinaria en Ingeniería y Tecnologías Avanzadas, Instituto Politécnico Nacional, Av. IPN No. 2580, L. Ticomán, Ciudad de México 07340, Mexico; ilopezr0600@alumno.ipn.mx; 2 Tecnológico Nacional de México, Tecnológico de Estudios Superiores de Ixtapaluca, Km. 7 Carretera Ixtapaluca-Coatepec S/N San Juan, Ixtapaluca, Estado de Mexico 56580, Mexico; cesar.rm@ixtapaluca.tecnm.mx; 3 Laboratorio de Investigación en Materiales Porosos, Instituto Politécnico Nacional-ESIQIE, Catálisis Ambiental y Química Fina, UPALM, Edificio 7 P. B., Zacatenco, C. P., Ciudad de México 07738, Mexico

**Keywords:** bipartite network, drug–disease network, active ingredients and diseases classification

## Abstract

The complexity of drug–disease interactions is a process that has been explained in terms of the need for new drugs and the increasing cost of drug development, among other factors. Over the last years, diverse approaches have been explored to understand drug–disease relationships. Here, we construct a bipartite graph in terms of active ingredients and diseases based on thoroughly classified data from a recognized pharmacological website. We find that the connectivities between drugs (outgoing links) and diseases (incoming links) follow approximately a stretched-exponential function with different fitting parameters; for drugs, it is between exponential and power law functions, while for diseases, the behavior is purely exponential. The network projections, onto either drugs or diseases, reveal that the co-ocurrence of drugs (diseases) in common target diseases (drugs) lead to the appearance of connected components, which varies as the threshold number of common target diseases (drugs) is increased. The corresponding projections built from randomized versions of the original bipartite networks are considered to evaluate the differences. The heterogeneity of association at group level between active ingredients and diseases is evaluated in terms of the Shannon entropy and algorithmic complexity, revealing that higher levels of diversity are present for diseases compared to drugs. Finally, the robustness of the original bipartite network is evaluated in terms of most-connected nodes removal (direct attack) and random removal (random failures).

## 1. Introduction

The need for the development of new drugs, together with the scalating (rising) cost and the time consumed to make new treatments available are the current challenges being faced by modern medicine and the pharmaceutical industry [1,2]. In the medical area, doctors usually have a table of medications for the treatment of a specific disease, which enable doctors to decide on the best treatment option, based on evidence from a clinical evaluation. In past decades, new tools, such as network medicine, have substantially contributed to a better understanding of the complex interaction of sub-systems in medicine, ranging from the molecular to the organism and ecosystem levels [3,4,5,6,7,8,9,10]. From a complex network’s perspective, it is important to evaluate potential interactions that can arise from at least three perspectives: drug–disease interactions, disease–disease interactions and drug–drug interactions. The construction of the bipartite network represents a crucial step to obtain particular information of the two layers and their corresponding projections. More specifically, the topological properties of the original bipartite and projected networks are strongly related to the the patterns of connectivities in the bipartite network and thus, define the local and global organizations of the projected ones [11,12]. The most widely explored example of such bipartite structure are the scientific collaboration networks, where nodes are authors or institutions, and a link exists if two authors have published an article together [13,14,15].

Understanding the role of drugs (active ingredients) and diseases is important for the characterization of the drug (or disease) “distances” in a general map, and eventually for drug re-purposing. In the context of biological networks, diverse approaches have been proposed for the discovery of new targets, characterization of the mechanism of action, identification of drug repurposing strategies, and for prediction of drug safety and toxicity. In particular, it has been observed that drugs often target regions and pathways that are shared across multiple conditions [16]. In a recent application of neural networks on graphs, a multi-layer representation of protein–protein, drug–protein, and drug–drug (with links representing side effects) interaction networks were used to predict side effects with improved performance over previous methods [17]. Despite the fact that various studies have explored the complex association between drugs and diseases, it is important to note that this characterization requires large-scale analyses that cover the various scales, from the molecular level to the information of comorbidities in patients [7,8,9,10,18,19,20,21,22,23]. In this work, we present a study of the network properties of the drug–illness interaction based on data from a recognized online pharmacological handbook [24]. The drug and illness degrees can be well described by a stretched-exponential function with different fitting parameters. This study reveals that projected networks exhibit noticeable differences in some network metrics when a threshold degree value is considered to link two nodes in the projection. The heterogeneity of the association at the group level between active ingredients and diseases is evaluated in terms of the Shannon entropy and algorithmic complexity. We find that a higher diversity is present for diseases, compared to drugs, and the complexity of the bipartite structure changes as the threshold degree is increased. Additionally, we perform an evaluation of the robustness of the bipartite network by considering two strategies of eliminating either fractions of the most connected or randomly selected nodes, and monitor the fragmentation of the projected networks. The robustness analysis offers a simple way to evaluate changes in the structure of the projected networks. The paper is organized as follows. In Section 2, a brief description of the drug–illness dataset is presented. The main results of the network properties are described in Section 3. Finally, some concluding remarks are provided in Section 4.

## 2. Data and Methods

### 2.1. Pharmacological Dataset

**Figure 1:**
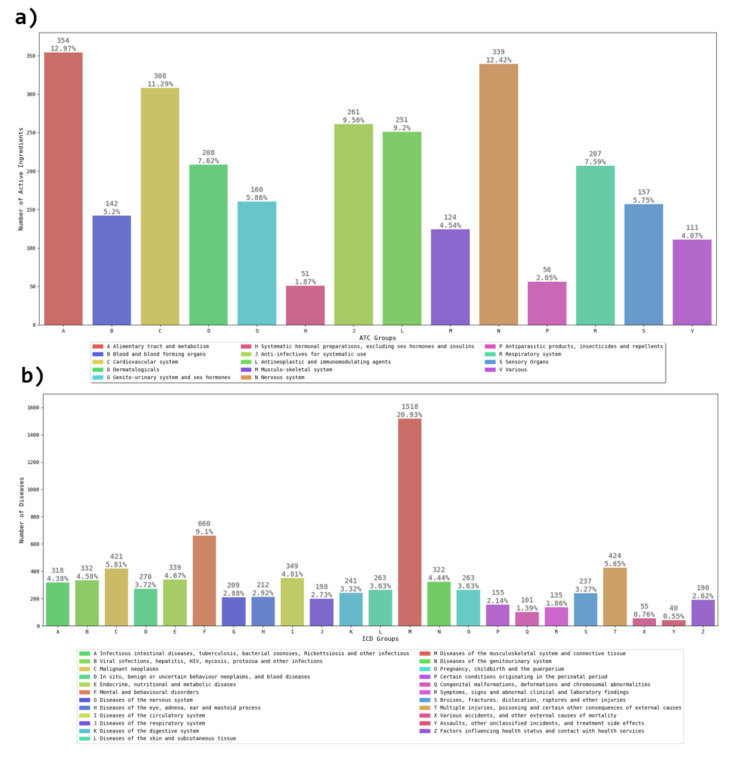
(**a**) Number of active substances that belong to a specific group, according to the Anatomical Therapeutic Chemical Classification System (ATC). At the top level, there are 14 main anatomical/pharmacological groups. We observe that groups A, N and C concentrate around 36% of the active substances. (**b**) Number of diseases per group, according to the International Classification of Diseases (ICD). At the top level, the number of main groups is 23. The group M contains 20% of the diseases.

Vademecum (VDM) is a well-recognized online pharmacological handbook, which includes advertising of medicines and prescriptions of pharmaceutical specialties addressed to health professionals [24]. Our group designed a database, based on the information provided by VDM, in order to have well-classified information about the roles of active pharmaceutical ingredients, diseases and side effects [25]. Our VDM dataset consists of tables of active pharmaceutical ingredients and diseases, where categorical groupings, such as Anatomical Therapeutic Chemical Classification System (ATC) and International Classification of Diseases (ICD), are assigned to the records [25]. The ATC Classification System classifies the active ingredients of drugs, according to the organ on which they act; the ICD is a classification system that provides a system of diagnostic codes for classifying diseases [26]. Specifically, ATC’s categorizations by anatomically therapeutic chemicals leads to a detailed classification of active substances, which, at its first level, has fourteen main anatomical/pharmacological groups (see Figure 1a). Similarly, ICD’s categorizations, at the top level, lead to twenty-three main groups (see Figure 1b). The usefulness of having the information with this level of classification is that by relating the anatomical levels from ATC and ICD codes, it is possible to identify that the anatomical name group (ATC) really fits with the organs or systems where the active ingredient should have an effect, for instance, the relationship N–G; here, the letter N from ATC is associated to substances which are created to help the nervous system, and the letter G from ICD refers to the diseases related to the nervous system.

We extracted from the VDM database drug–disease interactions resulting in a bipartite network, which consists of 2729 active ingredients targeting 7252 diseases. This resultant bipartite structure contains 9981 interactions.

### 2.2. Network Metrics

A bipartite network *G* can be defined as G=(A,D,E), where *A* and *D* are two disjoint sets of nodes, and *E* represents the set of edges. For the purpose of our study, we consider that set *A* is composed of active ingredient nodes $A_{1},A_{2},\cdots ,A_{n}$, and set *D* by $D_{1},D_{2},\cdots ,D_{m}$. The set of edges *E* contains the information if an active ingredient $A_{i}$ targets the disease $D_{j}$. For this reason, the edges exiting from an $A_{i}$ are described as outgoing, while the edges entering the node $D_{j}$ are denoted as incoming. We list some basic network metrics for the bipartite network and their projections [27]:

- Density (ρ): the density of a network is defined as follows:
  (1) $$\rho =\frac{g}{g_{max}},$$
  where *g* is the number of actual connections and $g_{max}=\left(r\left(r-1\right)\right)/2$ is the maximum number of edges with r=n and r=m, the number of nodes for drug and disease networks, respectively. Notice that for a bipartite network, the normalization term is given by $g_{max}=n\cdot m$.
  A value of ρ close to 1 denotes an almost complete graph, while ρ close to 0 indicates a poorly connected network.
- Shortest path length (*ℓ*): represents the shortest path between two nodes, i.e., a path with the minimum number of edges.
- Clustering coefficient ($C_{i}$): measures the degree of transitivity in connectivity amongst the nearest neighbors of a node *i* [27]:
  (2) $$C_{i}=\frac{2E_{i}}{k_{i}\left(k_{i}-1\right)},$$
  where $E_{i}$ is the number of links between the $k_{i}$ neighbors of the node *i*. The average clustering is the mean value from all nodes.
- Assortative mixing coefficient by degree ($A_{r}$): measure associated to the tendency frequently observed in networks, where nodes with a large number of neighbors are connected to other nodes with many (or a few) connections [27]. Formally, the coefficient is given by the following:
  (3) $$A_{r}=\frac{\sum_{ij}A_{ij}\left(k_{i}-\mu \right)\left(k_{j}-\mu \right)}{\sum_{ij}A_{ij}{\left(k_{i}-\mu \right)}^{2}},$$
  where $\mu =\frac{\sum_{ij}A_{ij}k_{i}}{\sum_{ij}A_{ij}}$. For perfectly assortative networks, the coefficient reaches a maximum value of 1, whereas a minimum value of −1 is observed for perfectly disassortative ones.

## 3. Results

### 3.1. Network Analysis

**Figure 2:**
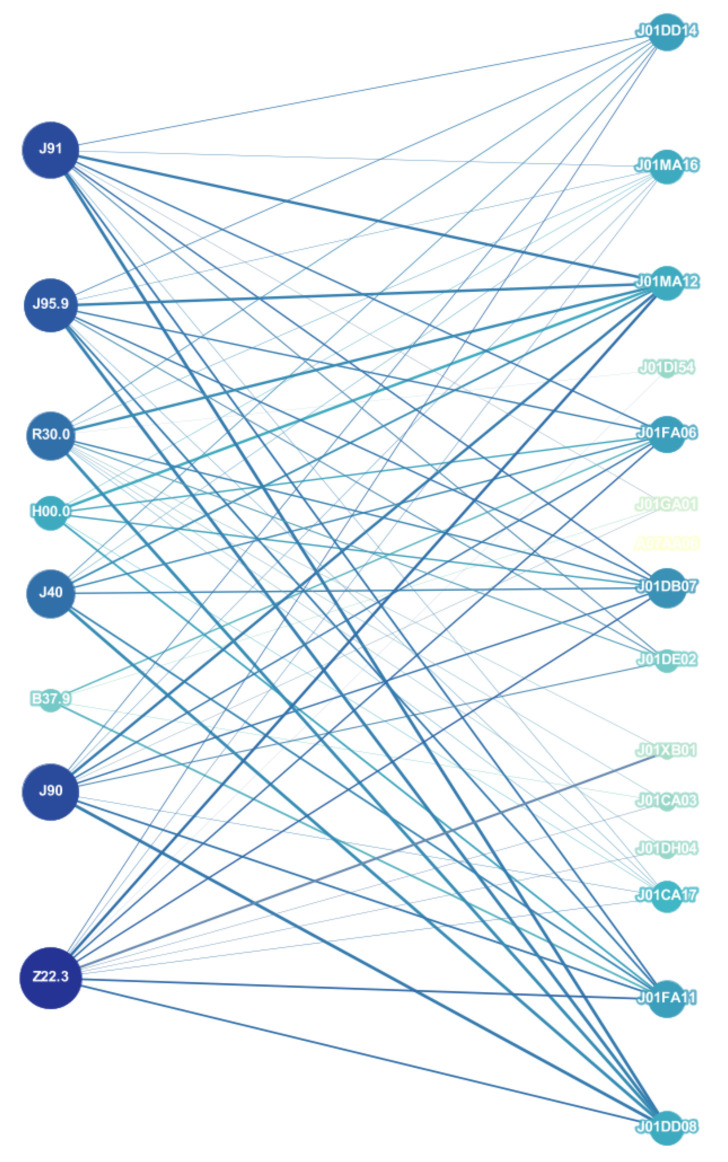
Representative subgraph of drug–disease bipartite network. Here, nodes on the left represent diseases (ICD) and the ones on the right denote active ingredients (ATC).

**Figure 3:**
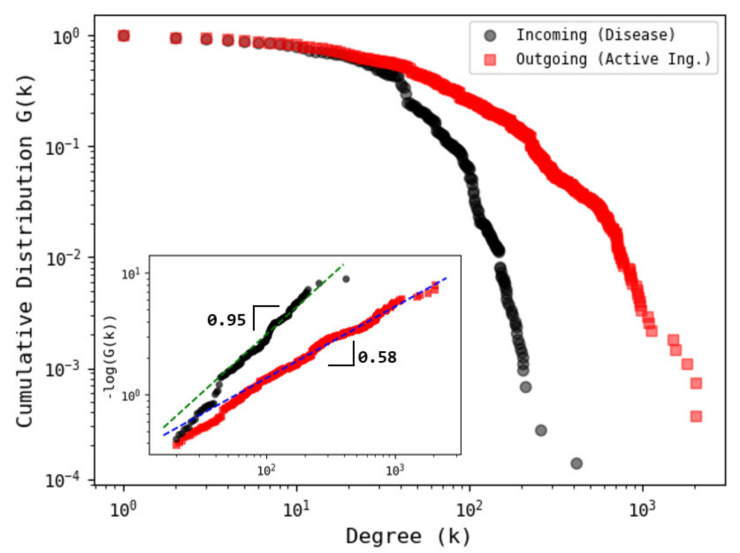
Cumulative degree distribution of ATC–ICD bipartite network. (Main) The corresponding distributions for incoming (disease) and outgoing (active ingredients) degrees are depicted. For diseases, the connectivities exhibit a rapid decay with exponential behavior, while for active ingredients, the data display a larger tail, following a stretched exponential function. (Inset) Log–log plot of −logG(k) vs. degree *k* for which a stretched exponential function appears as a straight line. Here, the slopes represent the value of the parameter *b*. We find that for incoming degrees b≈0.9, indicating that the distribution is very close to a pure exponential behavior, while for outgoing degrees, the value b≈0.5 points out that the behavior is stretched exponentially.

For this study, we considered the ATC–ICD association, using data from the VDM database. Although there are other available sources, our database contains a more detailed classification in terms of codes widely accepted by worldwide health agencies, such as the WHO. Our procedure allows us to identify that relationships between one ATC code and one ICD code can be very diverse, i.e., one ATC code points to various ICD codes (see Figure 2). We proceed to construct a bipartite network in which an active ingredient and a disease are linked if the disease is listed as a target of the active ingredient (see Figure 2). First, we explore some network properties such as the degree distribution. Figure 3 shows the behavior of the survival cumulative distribution of the connectivities for active ingredients (outgoing links) and diseases (incoming links). We observe that the distributions approximately follow a stretched exponential function of the general form $G\left(k\right)∼e^{-\alpha k^{\beta }}$ with α and β two fitting parameters.

In order to obtain a good estimation of the parameters, we construct the log−log plane of −log(G(k)) vs. *k*, where the stretched exponential functions adopts a linear behavior, the slope equals the parameter β, and the vertical intercept represents the value of the parameter α. We recall that the stretched exponential function is more skewed than the purely exponential one (β=1), but less skewed than a power law distribution (with a fat tail as β→0). Moreover, the characteristic scale (degree) associated to the stretched exponential distribution is given by $<k>=\frac{\alpha^{-1/\beta }}{\beta }\Gamma \left(1/\beta \right)$, with Γ being the Gamma function. Note that for β=1, the mean value of an exponential function is recovered. As shown in the inset of Figure 3, both distributions can be well approximated by a stretched exponential function with β=0.581±0.003, α=0.091±0.002 for active ingredients and β=0.951±0.018, α=0.037±0.003 for diseases. Moreover, for active ingredients, we obtain <k>=90.89, while for diseases, we obtain <k>=30.99, confirming the fast decay of the connectivities for incoming degrees (diseases). Our results provide evidence of a stretched exponential scaling, which indicates that the connectivities for outgoing links are found to lie somewhere between a power law and an exponential distribution, while the incoming degrees are close to the exponential behavior. These findings pointed out that the heterogeneity in the connectivities is higher for outgoing links (ATC), compared to the incoming degrees (ICD), reinforcing the idea of the effect of multi-purposing frequently found in drugs [28,29].

**Figure 4:**
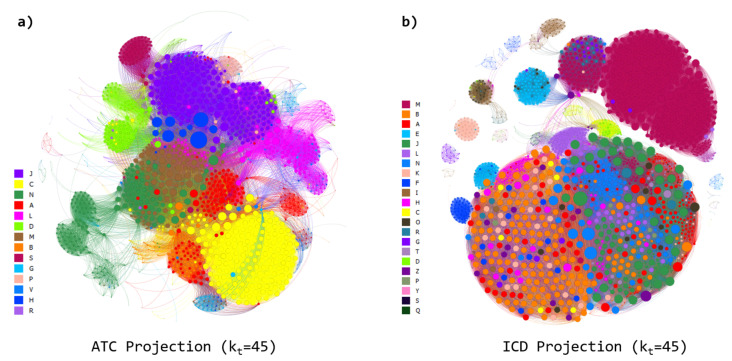
(**a**) Representation of the active-substances (ATC) projected network for a threshold degree $k_{t}=45$. For visualization purposes, the Fruchterman–Reignold [30] layout algorithm is used to enhance the proximity between nodes with same categorical group. (**b**) As in (**a**) but for the disease (ICD) projected network. The color of the nodes correspond to the group to which they belong.

Next, we generate two network projections: the active ingredient (ATC) network and the disease (ICD) network (see Figure 4). In the ATC network, nodes are active ingredients, and two active ingredients are connected to each other if they share at least one disease. In the ICD network, nodes are diseases, and two diseases are connected if they are targeted by at least one common active ingredient.

The main properties of the bipartite and the projected networks are shown in Table 1. The density in the bipartite network is quite small, compared to the corresponding density in the projections either on ATCs or ICDs, indicating that the co-occurrence phenomena are very frequent in both layers and is especially high for diseases. Both projected networks have similar average clustering coefficients and shortest path lengths. These results indicate that either substances or diseases have closer relationships with other nodes, due to the high probability of forming triangles, and the networks exhibit the small-world property [31]. Moreover, the disease network exhibits a positive value for the assortativity coefficient, while a very small (negative) value is observed for the active ingredient network, indicating that ICDs tend to be connected with other diseases with similar degree values; this is not observed for ATCs.

In order to evaluate the effect of the heterogeneity in the connectivity patterns of the bipartite network, we calculate some additional network properties of the projected ones. A threshold degree $k_{t}$ is considered as a condition for connecting two nodes (either active ingredients or diseases). The corresponding ATC and ICD projected networks for the threshold degree $k_{t}=45$ are depicted in Figure 4a,b, respectively.

**Figure 5:**
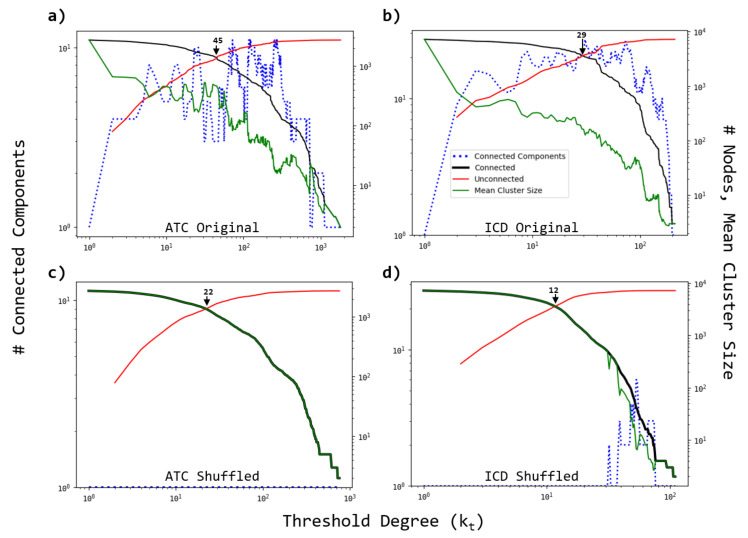
Behavior of the number of connected components, mean cluster size and number of connected (unconnected) nodes in terms of the threshold degree $k_{t}$ for (**a**) active substance (ATC) and (**b**) disease (ICD) projected networks. The threshold degrees $k_{t}=45$ and $k_{t}=29$ represent the values for which the number of connected and unconnected nodes reach the same value for ATC and ICD networks, respectively. (**c**,**d**) As in (**a**,**b**), but for the shuffled version of the bipartite network. We observe that the number of connected components remains equal to the unit as the threshold varies, except for a short interval in the ICD case, indicating that random associations promote the formation of a single component in the projections, even when the $k_{t}$ is increased. The results represent the average of 5 independent realizations.

Then the number of connected components (NCC), the number of connected (unconnected) nodes (NCN and NUN) and the mean cluster size (MCS) are calculated. Figure 5 shows the results of these calculations for ATC and ICD networks. For the ATC network (Figure 5a), the NCC increases for small values of $k_{t}$, then fluctuates around NCC ≈7 for intermediate threshold degrees ($10\leq k_{t}\leq 300$), and finally decreases for $k_{t}>300$. Notably, the mean size of the components (clusters) exhibits a high value (MCS = 321.63) for small and intermediate values $k_{t}<80$, and then decreases for large $k_{t}$ until reaching the limit value of 1. It is also evident that the number of connected (unconnected) nodes exhibits a monotonically increasing (decreasing) behavior as $k_{t}$ increases, revealing that there is a threshold value $k_{t}^{*}=45$, which separates two regions ($k_{t}<k_{t}^{*}$ and $k_{t}>k_{t}^{*}$) for which the mean size of the clusters slowly decreases ($k_{t}<k_{t}^{*}$), and then following by a rapid decreasing for larger threshold values ($k_{t}>k_{t}^{*}$).

For the ICD network (Figure 5b), we observe that the NCC exhibits an approximately convex behavior with higher values for intermediate threshold degrees $10\leq k_{t}\leq 80$, indicating that the patterns of association between ATCs and ICDs are markedly directed toward specific groups of ICDs, i.e., as $k_{t}$ increases, the appearance of connected components of ICD nodes which do not share links with other groups is more likely. We also observe that the corresponding curves of NCN and NUN intersect at the threshold degree $k_{t}^{*}=29$. In order to compare with a random version of the original bipartite network, we shuffle the outgoing and incoming links while preserving the degree sequence. The corresponding profiles of NCC, NCN, NUN and MCS for the projected networks are shown in Figure 5c,d. We observe that for both projections, the original profiles of the NCC changed to the unitary value independent of $k_{t}$, except for a small interval in the ICD network. We also notice that the crossover scale ($k_{t}^{*}$) for which the curves of NCN and NUN intersect each other is shifted to the left for both cases.

**Figure 6:**
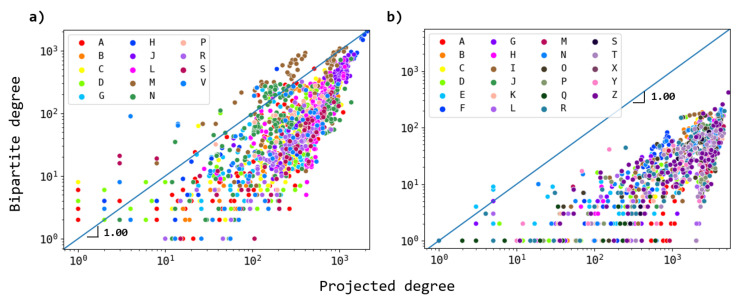
Scatter plot of node’s bipartite degree vs. node’s projected degree for the drug–disease network. (**a**) The ATC projection, where colors represent the ATC group to which each active ingredient belongs. While the majority of the nodes tend to increase their connectivities when projected, there are certain nodes from specific groups that, in fact, reduce their degree (located above the identity blue line). (**b**) As in (**a**) but for the ICD projection. Most of the nodes tend to notoriously increase their degree.

We further explore the changes in the connectivities of ATC and ICD nodes in the projected networks with respect to the corresponding node degrees in the bipartite network. In Figure 6, the behavior of these patterns’ connectivity is shown. For ATCs (Figure 6a), we observe that most of the groups tend to be below the identity line, meaning that for most of them, the active ingredients target common diseases, and less diversity is present. For ICDs (Figure 6b), it is found that most of the nodes notoriously increase their connectivities when they are projected, meaning that diseases tend to be more redundant in terms of connections to well-connected ATC nodes.

**Figure 7:**
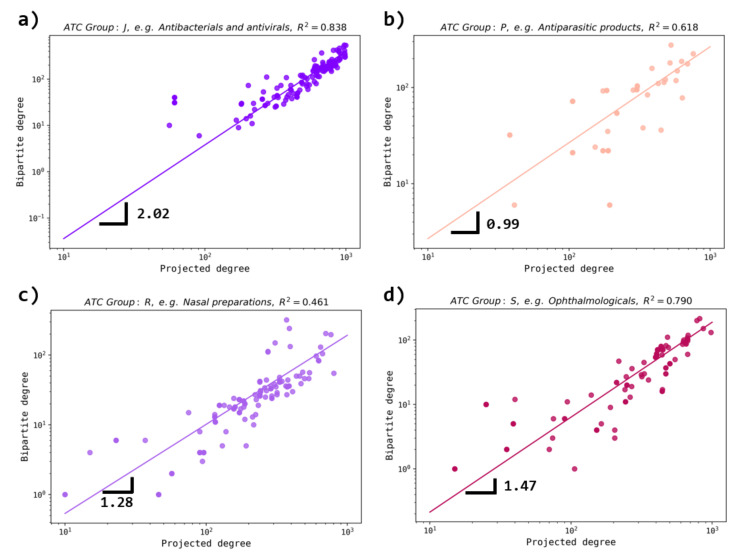
Behavior of the bipartite degree vs. projected degree for certain ATC groups. The cases of (**a**) group J (antibacterials and antivirals), (**b**) group P (antiparasitic products), (**c**) group R (nasal preparations) and (**d**) group S (ophthalmologicals) are depicted. The fitting region is $k>10^{2}$ for all cases. We observe that for groups J, R, and S, a superlinear behavior is present, while a linear behavior is observed for group P. Here, an exponent bigger than 1 indicates that for the fitting degree interval, the bipartite degree grows faster than their corresponding projected degree, i.e., the degrees of highly connected nodes in the bipartite network tend to have higher values than one corresponding to the projected network.

With a detailed inspection and the application of a power law fit, it is revealed (see Figure 7) that for ATC groups, such as J (antibacterials and antivirals), R (nasal preparations) and S (ophthalmologicals), the diversity is smaller compared to such cases as P (anti-parasitic products). The scaling exponent values for some ATC groups align with medical information about the wide range of diseases caused by parasites [32] for which drugs (with high diversity in targets) of group P are indicated, while the antibacterial and antiviral, ophthalmologic and nasal preparations groups do not follow this high diversity rate [33,34].

**Figure 8:**
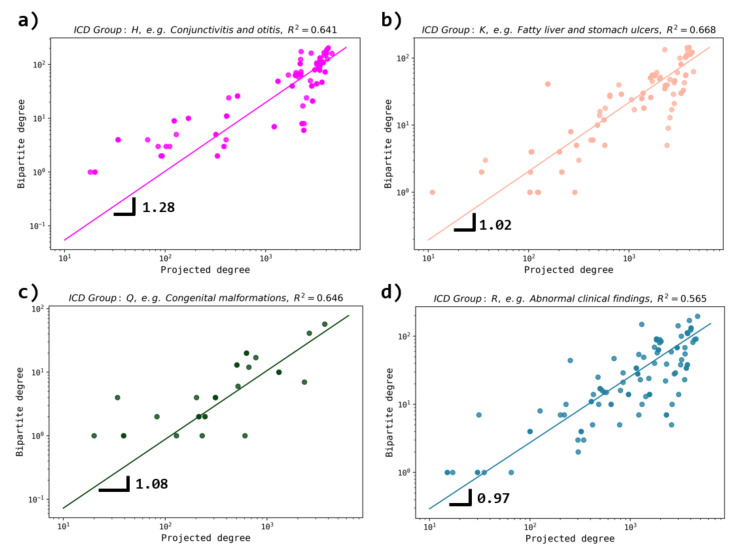
Behavior of the bipartite degree vs. projected degree for certain ICD groups. The cases of groups (**a**) H, (**b**) K, (**c**) Q and (**d**) R are depicted. The fitting region is $k>10^{2}$ for all cases. We observe that for all groups, a linear behavior is present. Here, an exponent close to 1 indicates that both degrees increase similarly.

On the other hand (Figure 8), for ICD nodes, most of the groups tend to exhibit a similar trend in the growth of degrees, i.e., diseases are associated with other diseases through drugs with similar rates and high diversity [29].

### 3.2. Shannon’s Entropy and Algorithmic Complexity

Next, to further describe the heterogeneity of group-level associations between ATCs and ICDs, we use two measures: Shannon entropy and algorithmic complexity [35,36,37]. The diversity index is calculated by means of the Shannon entropy $S_{E}=-\Sigma_{i}p_{i}\log p_{i}$, where $p_{i}$ represents the probability of a given ATC or ICD group [35]. The algorithmic complexity is based on Kolmogorov’s definition of complexity, which measures the amount of information needed to describe an object or system [36]. In this sense, systems represented by binary strings (including two-dimensional ones) can be well characterized by a (shortest) description of the regularities or symmetries it exhibits [38,39,40,41]. In many real cases, the estimation of the Kolmogorov complexity is not possible, and alternative methods have been proposed to estimate this measure, such as the Block Decomposition Method (BDM) [38]. Briefly, BDM considers the decomposition of the objects as smaller parts for which the algorithmic complexity can be estimated by an auxiliary method, such as the Coding Theorem Method [38].

**Figure 9:**
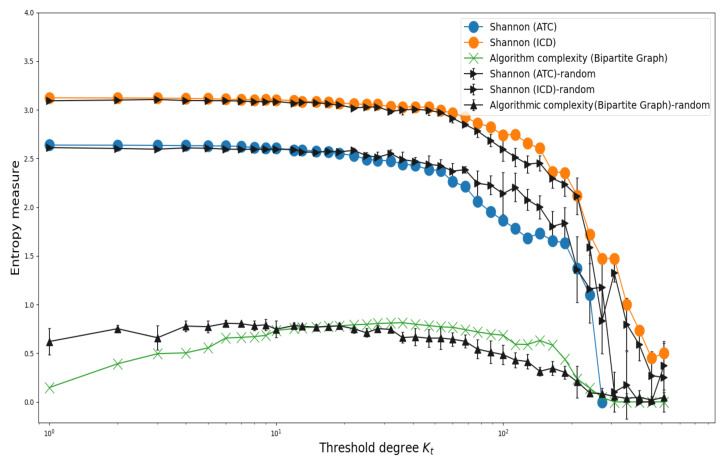
Behavior of entropy measures (Shannon and algorithmic complexity) for several threshold degree values. We observe that Shannon entropy is higher for ATCs compared to ICDs, and both decrease as the threshold degrees increases. In contrast, the complexity entropy obtained from BDM exhibits a convex behavior, suggesting that for certain interval degrees, the complexity displays higher values. The entropy values from randomized data are also presented. We observe that the Shannon entropy corresponding to the random configurations are close to the original data for both ATC and ICD, while a marked separation is observed for the case of the algorithmic complexity. Here, each value represents the average of 5 independent realizations, and the bars denote the standard deviation.

We evaluate the group-level connectivity diversity (Shannon entropy) between groups, which is defined as the number of different ICD (ATC) groups to which an ATC (ICD) group is associated in the bipartite graph, i.e., the extent of diversity in terms of the number of different groups of ATC (ICD) shared in common. Moreover, in order to obtain an estimate of the complexity of the bipartite structure, we apply the BDM to binarized adjacency matrices of the bipartite graph, which is constructed considering the threshold degree $k_{t}$ (see previous section for details). The calculations of the Shannon entropy for ATC and ICD groups and the algorithmic complexity as a function of the threshold degree $k_{t}$ are shown in Figure 9. We observe that $S_{E}$ is higher for ICDs, compared to ATCs, while exhibiting a decrease as the threshold degree increases, i.e., the heterogeneity in the connections is reduced by imposing a higher linkage requirement for either ATCs or ICDs. In contrast, the algorithmic complexity obtained from BDM exhibits a convex behavior, i.e., for small values of $k_{t}$, the complexity is relatively small and grows for intermediate values of the threshold degree value until it finally decreases for large values, revealing that the most heterogeneous configurations are reached for intermediate values of $k_{t}$. Additionally shown in Figure 9 are the values of the entropic measures for the randomized version of the bipartite network. It is observed that the values $S_{E}$ from randomized configurations are similar to the values of the original ones, except for the intermediate threshold values, where random data surpass ATC data, while the opposite is true for ICDs. For the algorithmic complexity, the randomized values exceed the original data for thresholds $k_{t}\leq 10$, and then this behavior is reversed for intermediate values of $k_{t}$.

### 3.3. Robustness of the Networks

In order to test the robustness of the ATC-ICD connectivity patterns, we measure the effects of directed attacks and random failures. Specifically, we consider the following procedure:

- A fraction of either ATC or ICD nodes are removed from the original and the randomized bipartite networks. Two strategies are considered. The nodes to be removed are either chosen as the most connected ones (directed attacks), or at random (random failures).
- The average cluster size is evaluated to evaluate the effect of the node’s removal.
- The process is repeated for several fractions of removed nodes.

**Figure 10:**
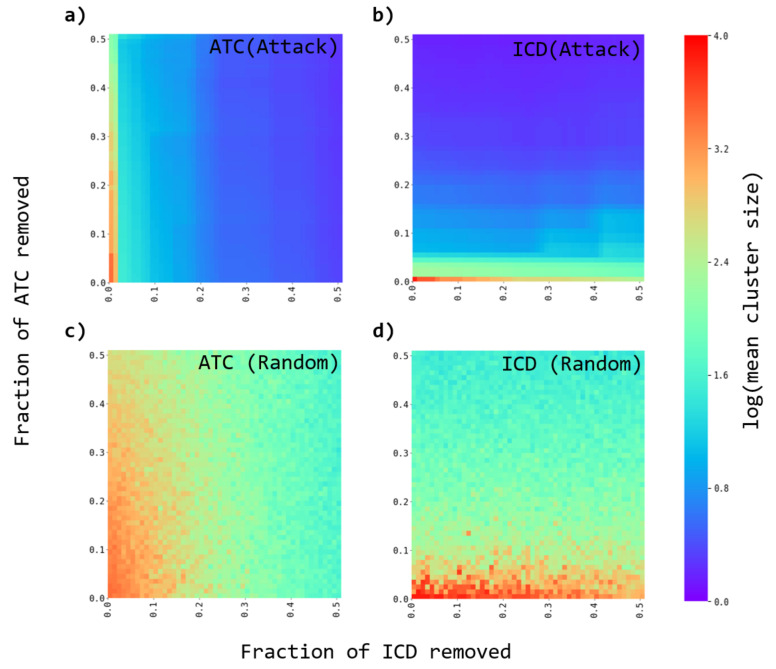
Average cluster size profiles for the projected networks under directed attacks and random failures. Panels (**a**,**b**) show the cases of the ACT and ICD projected networks after attacks, respectively. Panels (**c**,**d**) show the cases of the effect of random failures on ATC and ICD projected networks, respectively. We observe that the average fragment size in the ATC (ICD) projection is more sensitive to attacks on ICD (ATC) nodes. In contrast, a slower decay of the average fragment size is present for random removals either for ATC or ICD. Note that a logarithmic color scale is used to resolve the rapid drop in the average cluster size.

In Figure 10, the effect of directed attacks and random failures is depicted. It is observed that the average cluster size for directed attacks exhibits different patterns when both projections are compared (Figure 10a,b), either ATC or ICD, indicating that the removal of a small fraction of either ATC or ICD nodes leads to noticeable changes in the fragmentation (notice that a logarithmic scale is used in the color bar scale). More specifically, a slower decay of the cluster sizes is present for the ICD network compared to the ATC, as the fraction of most connected ATC (or ICD) nodes removed is increased. The fragmentation is clearly more sensitive to attacks in the opposite layer (either ATC or ICD), as a rapid drop is observed when attacks occur on ICDs or ATCs. When ATC and ICD projections are compared with their corresponding randomly attacked networks, we see that slower decays are present for both projections, confirming that the networks are less sensitive to random failures, either on ATCs or ICDs.

### 3.4. Comparison with Other Database

**Figure 11:**
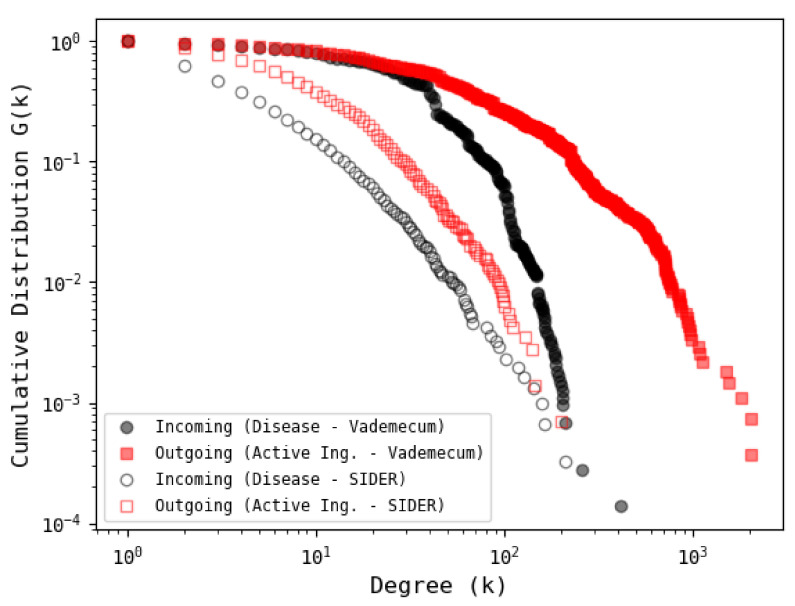
Cumulative degree distribution of ATC–ICD bipartite network from SIDER and VDM databases. Both outgoing and incoming SIDER distributions exhibit stretched exponential behavior with faster decays compared to VDM distributions. See supporting information online at *FigShare* https://figshare.com/s/0fbf899859bf732baa0b (accessed on 15 June 2021) for further details of the comparison between the two networks and a high resolution version of this figure.

In order to have a comparison with another database, the SIDER database [42], which has a moderate number of records, is considered. The results of the cumulative distribution corresponding to active ingredients and diseases are shown in Figure 11. In general, it is observed that the distributions follow a stretched exponential type behavior where the tails for the VDM network are longer compared to those of SIDER, related to the relatively low number of records in SIDER. For further details of the comparison between the two networks, consult the information available online through *FigShare* (https://figshare.com/s/0fbf899859bf732baa0b (accessed on 15 June 2021)).

## 4. Conclusions

We have presented an analysis of the drug–disease interaction network. The need for new drugs together with their cost implications have contributed to the formation of a complex drug–disease interaction, the understanding of which is important for evaluating indirect interactions between active substances and the proximity between diseases. Our results on outgoing and incoming links have confirmed that drugs and diseases follow different pattern of connectivities, where drugs follow a stretched exponential distribution, while diseases are close to an exponential function. This means that there are few drugs that target several diseases, while an average number of targets (diseases) have many active ingredients prescribed. The analysis of the projected networks has indicated that the ATC and ICD networks exhibit different topological properties and fragmentation profiles, which are markedly different when the links in the bipartite network are randomly rewired. The net effect of rewiring the bipartite network is to reduce the level of redundancy (more diversification in the associations), giving rise to the emergence of a single component in the projections, either on ATC or ICD. We also showed that the changes in the node’s degree during the projections characterize the tendency of ATC to share diseases with other well-connected ATCs. This behavior is more pronounced from the ICD perspective, as the majority of diseases notoriously increase their connectivities, giving rise to a very dense network. The use of entropic measures, such as Shannon entropy and algorithmic complexity, has provided a way to assess the level of heterogeneity in associations at the anatomical group level and allowed us to identify the effects of association intensity on diversity in emergent configurations. We also have evaluated the effect of different node removal strategies (attacks and random failures) to perform projections onto ATCs and ICDs. Important differences in the fragmentation of the projected networks were observed, suggesting that inactivating or dispensing with a few highly connected drugs would greatly separate the remaining diseases. This study has some limitations mainly related to the number of records and interactions. Although the database analyzed (VDM) contains a significant number of active ingredients and diseases, other databases, such as DrugBank [43] and DrugCentral [44], report different counts for the active ingredients (and diseases) when classified into anatomical groups. The correspondence between the different databases needs to be evaluated in order to have a more appropriate classification, and to determine the general properties of the networks that are representative of all databases. Another important consideration has to do with the fact that the VDM database is focused on the Spanish-speaking market, which makes it naturally different from others based in other regions of the world. Finally, the results reported in this work represent only an initial look at the complex drug–disease interactions from the perspective of network–pharmacological classification.

## Figures and Tables

**Table 1:** Representative network metrics of the drug–disease network, the projected drug network and the disease network. For all the networks, we listed the number of nodes, the number of edges, the mean degree and the density. The shortest path length, clustering and the assortativity index are listed for the drug and disease networks.

Metric	Bipartite	Disease	Active Ingredient
Number of nodes	9981	7252	2729
Number of edges	260,995	6,188,810	454,164
Mean degree	52.29	1706.78	332.84
Density	0.005	0.235	0.122
Average shortest path length	-	1.84	1.99
Average clustering	-	0.724	0.735
Assortativity	-	0.217	$-3.53\;(10^{-2})$

## Data Availability

Not applicable.
